# Association between age at first reported e‐cigarette use and subsequent regular e‐cigarette, ever cigarette and regular cigarette use

**DOI:** 10.1111/add.15386

**Published:** 2021-01-12

**Authors:** Mark Conner, Sarah Grogan, Ruth Simms‐Ellis, Lisa Cowap, Christopher J. Armitage, Robert West, Anna‐Marie Marshall, Kamran Siddiqi

**Affiliations:** ^1^ School of Psychology University of Leeds Leeds UK; ^2^ Department of Psychology Manchester Metropolitan University Manchester UK; ^3^ Centre for Psychological Research, Science Centre Staffordshire University Stoke‐on‐Trent UK; ^4^ Manchester Centre for Health Psychology, Division of Psychology and Mental Health, Manchester Academic Health Science Centre University of Manchester Manchester UK; ^5^ Institute of Health Sciences University of Leeds Leeds UK; ^6^ Department of Health Sciences University of York York UK

**Keywords:** Adolescents, E‐cigarettes, electronic nicotine delivery systems, harm reduction, intervention, smoking

## Abstract

**Background and aims:**

Association of electronic cigarette use and subsequent smoking has received considerable attention, although age of first use has not. This study tested differences in regular (e‐cigarettes, cigarettes) and ever (cigarettes) use between e‐cigarette user groups: early versus never users, late versus never users, early versus late users and effects of controlling for covariates.

**Design:**

Prospective study with 12‐ and 24‐month follow‐up of e‐cigarette/cigarette ever/regular use with data from an intervention.

**Setting:**

Forty‐five schools in England (Staffordshire and Yorkshire).

**Participants:**

Never smokers (3289 13–14‐year‐olds) who were part of a cluster randomized controlled trial.

**Measurements:**

The sample was divided into groups of e‐cigarette users: early users (at 13–14 years), late users (at 14–15 years) and never users (at 13–14 and 14–15 years). Dependent variables were self‐reported regular e‐cigarette and cigarette use and ever cigarette use at 15–16 years. Covariates were assessed.

**Findings:**

Early and late users compared with never users were significantly more likely to be regular e‐cigarette users [early: odds ratio (OR) = 9.42, 95% confidence interval (CI) = 5.38, 16.49, *P* < 0.001; late: OR = 6.89, 95% CI = 4.11, 11.54, *P* < 0.001], ever cigarette users (early: OR = 7.96, 95% CI = 6.02, 10.53, *P* < 0.001; late: OR = 5.13, 95% CI = 3.85, 6.84, *P* < 0.001) and regular cigarette users (early: OR = 7.80, 95% CI = 3.99, 15.27, *P* < 0.001; late: OR = 4.34, 95% CI = 1.93, 9.77, *P* < 0.001) at age 15–16 years. Late users compared with early users had significantly lower rates of ever use of cigarettes at 15–16 years (OR = 0.48, 95% CI = 0.35, 0.66, *P* < 0.001), although this difference was non‐significant at 12 months after first use of e‐cigarettes (OR = 0.89, 95% CI = 0.64, 1.25, *P* = 0.498). Controlling for covariates did not change the findings.

**Conclusions:**

Adolescents in England who report using e‐cigarettes at age 13–14 years have higher rates of subsequently initiating cigarette use than adolescents who report using e‐cigarettes at age 14–15 years, a difference that may be attributable to a longer period of time to initiate cigarette use in former group.

## Introduction

An increasing number of studies have assessed the impact of using e‐cigarettes on subsequent initiation of smoking in adolescents. Various US [1, 2, 3, 4] and UK [5, 6, 7] studies show e‐cigarette use by adolescents to be positively associated with initiation of cigarette use 12–24 months later in 14–16‐year‐olds. The evidence in relation to e‐cigarette use and becoming a regular user of cigarettes is more mixed [7, 8]. Few of the studies examining the relationship between e‐cigarette use and subsequent smoking have examined the role of age of uptake (first reported use) of e‐cigarettes. McCabe *et al*. [9] is one exception. They reported data comparing US adolescents who began using e‐cigarettes in the ninth grade (14–15 years or earlier) versus the 12th grade (17–18 years). Those adolescents who initiated e‐cigarette use earlier compared to later were significantly more likely to become ever users of cigarettes [adjusted odds ratio (aOR) = 2.83, 95% confidence interval (CI) = 1.06, 7.51] when high school seniors. More recently, Evans‐Polce and colleagues [10] show that age of first reported use of e‐cigarettes is changing in US adolescents, while it appears to be remaining stable for first reported use of cigarettes, cigars and smokeless tobacco. In particular, between 2014 and 2018 the proportion of ever users of e‐cigarettes at 14 years or younger increased from 8.8 to 28.6%. It is notable that the cross‐sectional data reported by Evans‐Polce *et al*. [10] do not indicate that earlier uptake of e‐cigarettes is being translated into earlier uptake of cigarettes or other tobacco‐containing products. The present research provides a further test of the impact of age of first reported use of e‐cigarettes (i.e. early versus late use) on subsequent ever cigarette use in a sample of UK adolescents. In addition, the present research provides a novel test of the impact of early versus late use of e‐cigarettes on subsequent regular cigarette use and regular e‐cigarette use.

The present data on e‐cigarette and cigarette use in UK adolescents were collected as part of a trial to test an intervention to prevent smoking initiation [11]. Previous reports have focused on never smokers aged 13–14 years who reported having used e‐cigarettes or not to predict initiation of cigarette smoking 12 months later when aged 14–15 years [5] and initiation and regular smoking 24 months later when aged 15–16 years [7]. We also collected data on use of e‐cigarettes at age 14–15 years that we have not published previously, and this has allowed us to compare groups of never smoking adolescents who first reported e‐cigarette use at different ages [early (13–14 years) versus late users (14–15 years)] in relation to their self‐reported regular e‐cigarette use, ever cigarette use and regular cigarette use at age 5–16 years. Such comparisons of early and late users confound age of first use of e‐cigarettes with time delay between first reporting e‐cigarette use and when outcomes are assessed (i.e. 24 months in early users versus 12 months in late users). To explore further the effect of time delay we also compared early and late users of e‐cigarettes at 12 months after first use (i.e. at age 14–15 years in early users versus 15–16 years in late users). Our analyses examined the effects of age of first reported use of e‐cigarettes when not controlling and controlling for various covariates of adolescent smoking.

Although our focus was on the comparison of early versus late first users of e‐cigarettes, groups of adolescents who had not used e‐cigarettes were also created to enable comparison. The never users comparison group for early users of e‐cigarettes were adolescents who were also never smokers at age 13–14 years and never users of e‐cigarettes at both 13–14 and 14–15 years. The never users comparison group for late users of e‐cigarettes were a subset of this group who additionally were never smokers at age 14–15 years. The user versus never user comparisons assessed the impact of e‐cigarette use on subsequent cigarette use.

The present study extends knowledge in this area in four important ways. First, this is the only study, to our knowledge, to examine effects of early versus late use of e‐cigarettes on regular e‐cigarette, ever cigarette use and regular cigarette use. Second, this study explored outcomes both at age 15–16 years and at 12 months after first reported use of e‐cigarettes, in order to help differentiate between the effects of age of initiation and the duration from first use of e‐cigarettes to assessment of outcome. Third, this study explored the effects of controlling for a number of covariates (gender, ethnicity, individual/school‐level socio‐economic status, friends and family smoking, impulsivity and intentions, attitudes, norms, perceived behavioural control and self‐efficacy in relation to smoking). Fourth, self‐reported measures of smoking at follow‐up were validated against objective smoking measures.

The current study had three specific aims: (1) to test for differences between early and never users of e‐cigarettes in subsequent regular use of e‐cigarettes, ever use of cigarettes and regular use of cigarettes; (2) to test for differences between late and never users of e‐cigarettes in subsequent regular use of e‐cigarettes, ever use of cigarettes and regular use of cigarettes; and (3) to test for differences between early and late users of e‐cigarettes in subsequent regular use of e‐cigarettes, ever use of cigarettes and regular use of cigarettes.

## Method

### Design

Data were collected as part of a pre‐registered, 4‐year cluster‐randomized controlled trial of a school‐based intervention to prevent smoking initiation [11, 12] using anti‐smoking messages and implementation intentions [13, 14, 15]. The study was conducted in 45 schools in England with adolescents initially aged 11–12 years. The analyses reported here were performed controlling for condition.

### Participants and procedures

The data reported here are from wave 3 (September–December 2014 in 13–14‐year‐olds), wave 4 (September–December 2015 in 14–15‐year‐olds) and wave 5 (September–December 2016 in 15–16‐year‐olds) of the trial when measures of e‐cigarette use were added to data collection. Only respondents reporting having never smoked a cigarette at wave 3 were analysed here (i.e. this is a *post‐hoc* analysis), although adolescents in both control and intervention conditions were included. Previous papers reported the impact of e‐cigarette use (wave 3) on smoking 12 months later (wave 4; control condition only) [5] and 24 months later (wave 5; control plus intervention conditions) [7]. The University of Leeds, UK (Faculty of Medicine) ethical review committee approved the study (reference 12–0155).

### Measures

#### Outcomes

Cigarette use was assessed using standardized measures [16] at each wave (i.e. time‐point); adolescents ticked one of: ‘I have never smoked; I have only tried smoking once; I used to smoke sometimes, but I never smoke cigarettes now; I sometimes smoke cigarettes now, but I don't smoke as many as one a week; I usually smoke between one and six cigarettes a week; and I usually smoke more than six cigarettes a week’. Only respondents marking the first response at wave 3 were retained for analysis. At waves 4 and 5 this measure was converted into outcome measures of ever smoked cigarettes (first response coded 0; other responses coded 1) and regularly smoked cigarettes (last two responses coded 1; other responses coded 0). The self‐reported smoking responses were validated against a measure of breath carbon monoxide (CO) levels (Micro+ Smokerlyzer^®^ CO Monitor; Bedfont Scientific Limited, Kent, UK), although we did not reclassify self‐report measures based on CO level. Such measures are reliable and valid ways of assessing regular cigarette smoking [17, 18] but not occasional smoking, due to the short half‐life (4–6 hours) of breath CO.

E‐cigarettes/vapourizers were described as ‘a tube that sometimes looks like a normal cigarette and has a glowing tip. They all puff a vapour that looks like smoke but unlike normal cigarettes, they don't burn tobacco’. Use of e‐cigarettes at each wave was tapped by one item [‘which ONE of the following is closest to describing your experience of e‐cigarettes or vapourisers?, I have never used them; I have tried them once or twice; I use them sometimes (more than once a month but less than once a week); I use them often (more than once a week’)]. This was converted into a measure of ever used e‐cigarettes (first response = 0; other responses = 1) and regularly used e‐cigarettes (last response coded 1; other responses coded 0). Ever used e‐cigarettes at waves 3 and 4 was used to classify respondents into different user groups (see below). Regularly used e‐cigarettes at waves 4 and 5 was used as an outcome measure.

#### Predictors

Other measures were assessed as covariates that might account for the relationship between e‐cigarette and cigarette use. Demographic variables analysed were gender (male = 0; female = 1), ethnicity (non‐white = 0, white = 1), family affluence (based on the four‐item Family Affluence scale [19]: scored 0–3, higher scores indicating greater affluence). Two school‐level measures were also assessed: percentage of children per school eligible for free school meals (< median 20% = 0; ≥ 20% = 1) [20]; condition (control = 0, intervention = 1).

Friends’ smoking was assessed using the question: ‘How many of your friends smoke?’—none of them; only a few; half and half; most but not all; all of them (none of them = 0; a few or more = 1). Family smoking was assessed using the question: ‘Who smokes in your family now? Tick all the people who smoke at the moment’, followed by a list of eight family members plus open‐ended response to specify additional family member (no family members = 0; one or more family members = 1). Friends and family smoking were assessed at wave 3. Impulsiveness was measured at wave 5 based on a five‐item impulsivity scale (dichotomized into low impulsivity = 0, high impulsivity = 1) [20].

Health cognitions about smoking [14] were assessed at wave 3 as the mean of multiple questions on five‐point scales: intention (three questions; e.g. ‘I plan not to smoke’; Cronbach's α = 0.90); attitude (seven questions; e.g. ‘For me, smoking would be… good–bad’; α = 0.87); perceived norms (five questions; e.g. ‘Most of my friends think… I should smoke–I should not smoke’; α = 0.79); perceived behavioural control (three questions; e.g. ‘I am confident I could resist smoking’, strongly disagree–strongly agree; α = 0.69); and self‐efficacy (six questions; e.g. ‘I can say no to smoking, even at school’, strongly disagree–strongly agree; α = 0.91). Questions were highly skewed towards negative views of smoking and so were dichotomized (negative views = 0; less negative views = 1).

### Data analysis

The analyses reported here were not pre‐registered, and as such the results reported should be considered exploratory. Missing data ranged from 0.0% (gender) to 1.1% (ethnicity) and 98% of the 3289 never smokers in the sample would have been available for analysis under the traditional listwise deletion method across variables. Data were missing due to item non‐response. As the level of missing values was low, missing at random was assumed to justify multiple imputation. Multiple imputation in SPSS generated five imputed data sets. Imputed values compared reasonably to observed values and results using listwise deletion were similar to multiple imputation, so imputed results are presented for all analyses. Adolescents were clustered by school and tests of differences between user groups (see below) controlled for clustering by school.

Based on responses to questions about ever use of e‐cigarettes and cigarettes at waves 3 and 4, different e‐cigarette user groups were created. Early users were defined as those who at wave 3 reported ever use of e‐cigarettes plus never use of cigarettes, i.e. they had started using e‐cigarettes but not cigarettes by age 13–14 years (*n* = 649). Late users were defined as those who at wave 3 reported never use of e‐cigarettes or cigarettes and at wave 4 reported ever use of e‐cigarettes plus never use of cigarettes, i.e. they had started using e‐cigarettes but not cigarettes by age 14–15 years (*n* = 419). Never users were defined as those who at both waves 3 and 4 reported never use of e‐cigarettes plus at wave 3 reported never use of cigarettes (*n* = 2221). A subgroup of never users (labelled never users who never smoked) was defined as those who reported at both waves 3 and 4 that they never used e‐cigarettes or cigarettes (*n* = 2172).

In the first set of analyses on the e‐cigarette user group, early and never users were compared on regular use of e‐cigarettes, ever use of cigarettes and regular use of cigarettes at waves 4 and 5. The tests at wave 4 represent a 12‐month follow‐up; the tests at wave 5 represent a 24‐month follow‐up. These analyses overlap with those previously reported from these data [5, 7]. In the second set of analyses on the e‐cigarette user group, late and never users who never smoked were compared on regular use of e‐cigarettes, ever use of cigarettes and regular use of cigarettes at wave 5 (i.e. a 12‐month follow‐up). These comparisons have not previously been reported. In the third set of analyses on the e‐cigarette user group, early and late users were compared regular use of e‐cigarettes, ever use of cigarettes and regular use of cigarettes at waves 4 and 5. Two comparisons between early and late users were possible here, although we focus upon the former: comparisons at a specific age point, i.e. wave 5 (i.e. when adolescents were aged 15–16 years); and comparisons on effects 12 months after e‐cigarette use first reported (i.e. wave 4 outcomes for early users; wave 5 outcomes for late users). It is worth noting that the time‐periods since reporting e‐cigarette use and age are confounded in these analyses. In the former analyses, time delay to outcome is different (early users: 24 months; late users: 12 months), although age when outcome is assessed is matched (15–16 years). In the latter analyses, time delay to outcome is matched (12 months), but age when outcome is assessed is different (early users: 14–15 years; late users: 15–16 years). Comparison of these analyses should give some indication of whether differences between early and late users are attributable to age of first reported use of e‐cigarettes or time delay from first use to outcome reporting.

IBM SPSS, version 26 or HLM, version 7 were used for the analyses. We assessed sample characteristics and validated cigarette smoking measures at waves 4 and 5 against breath CO levels (using logistic regressions). The main analysis assessed the frequencies of regular e‐cigarette use, ever use of cigarettes and regular cigarette use in each of the e‐cigarette user groups at wave 5 (and for some groups at wave 4). Analyses predicted regular e‐cigarette use, ever cigarette use and regular cigarette use (at wave 5) based on user group (model 1) plus covariates (model 2) using multi‐level logistic regression (Bernoulli model) that controlled for clustering by school. The covariates were included as potential explanations of the relationship between e‐cigarette use and later cigarette use (all were level 1 variables except free school meals and condition, which were level 2 variables). For comparison purposes, we also compared the early and late user groups at 12 months after first reported use (i.e. wave 4 outcomes for early users against wave 5 outcomes for late users). For group comparisons we report the odds ratio (OR), 95% CI and *P*‐value and examine the effects of controlling for covariates (based on the population average model with robust standard errors). The comparison between early and late users report these statistics for group with and without covariates and the −2 log‐likelihood to indicate model fit.

Full data are available from the first author.

## Results

### Sample description

Table 1 provides descriptive data on all measures for the full imputed sample. The sample of 3289 never smokers at age 13–14 years (Table 1) comprised 47.0% boys and 83.5% white individuals, who mainly had no friends who smoked (66.8%) but had one or more family member who smoked (61.3%). Health cognitions about smoking were all biased against smoking.

**Table 1:** Descriptive data for the sample (*n* = 3289)

		*n*	(%)
Sex	Boy	1546	(47.0%)
	Girl	1743	(53.0%)
Ethnicity	Non‐white	542	(16.5%)
	White	2747	(83.5%)
Family affluence^a^		2.72	(0.49)
Friend smokers	None	2196	(66.8%)
	A few or more	1093	(33.2%)
Family smokers	None	1272	(38.7%)
	One or more	2017	(61.3%)
Impulsivity	Low	1491	(45.3%)
	High	1798	(54.7%)
Intention	Low	3021	(91.9%)
	High	268	(8.1%)
Attitude	Low	2740	(83.3%)
	High	549	(16.7%)
Norms	Low	2859	(86.9%)
	High	430	(13.1%)
Perceived behavioural control	Low	2713	(82.5%)
	High	576	(17.5%)
Self‐efficacy	Low	2688	(81.7%)
	High	601	(18.3%)
Free school meals^b^	Low	22	(48.9%)
	High	23	(51.1%)
Condition^b^	Control	20	(44.4%)
	Intervention	25	(55.6%)

^a^ Mean and standard deviation for this variable

^b^ number of schools

Objectively assessed breath CO levels were predictive of being classified as a regular cigarette smoker at wave 4 (OR = 2.52, 95% CI = 1.54, 4.10, *P* < 0.001) and wave 5 (OR = 2.10, 95% CI = 1.71, 2.58, *P* < 0.001). Objectively assessed breath CO levels were predictive of being an ever cigarette smoker at wave 5 (OR = 1.12, 95% CI = 1.01, 1.24, *P* = 0.03) but not wave 4 (OR = 1.11, 95% CI = 0.94, 1.31, *P* = 0.23). However, it is worth noting that the median and range of breath CO levels were very similar for different categories of smoker at both waves.

### E‐cigarette user groups

Table 2 shows the numbers of adolescents reporting different levels of regular e‐cigarette use, ever cigarette use or regular cigarette use in the different e‐cigarette user groups at wave 4 (when aged 14–15 years) and wave 5 (when aged 15–16 years). At wave 4, rates of ever smoking were higher in early users compared to never users (Table 2, left‐hand panel). Logistic regression analyses that controlled for clustering confirmed these differences to be significant when not controlling for covariates (OR = 16.69, 95% CI = 12.45, 22.37, *P* < 0.001) and when controlling for covariates (OR = 1.39, 95% CI = 1.29, 1.50, *P* < 0.001). Rates of regular e‐cigarette and cigarette use were also higher in early users compared to never users at wave 4, although we did not test these differences in logistic regression, given the low numbers involved (Table 2). At wave 5, early users again had higher rates of regular e‐cigarette use, ever cigarette use and regular cigarette use compared to never users (Table 2, right‐hand panel). After controlling for clustering, each of these differences was significant when not controlling (regular e‐cigarette use: OR = 9.42, 95% CI = 5.38, 16.49, *P* < 0.001; ever cigarette use: OR = 7.96, 95% CI = 6.02, 10.53, *P* < 0.001; regular cigarette use: OR = 7.80, 95% CI = 3.99, 15.27, *P* < 0.001) and controlling (regular e‐cigarette use: OR = 1.21, 95% CI = 1.11, 1.31, *P* < 0.001; ever cigarette use: OR = 3.55, 95% CI = 2.82, 4.49, *P* < 0.001; regular cigarette use: OR = 1.25, 95% CI = 1.16, 1.34, *P* < 0.001) for covariates. These findings parallel our previously reported findings [5, 7].

**Table 2:** Frequency of e‐cigarette and cigarette use at different waves (ages) split by e‐cigarette user group

	Behaviour at wave 4 (age 14–15 years)			Behaviour at wave 5 (age 15–16 years)Behaviour at wave 5 (age 15–16 years)		
	E‐cigarette use	Cigarette use		E‐cigarette use	Cigarette use	
Group	Regular	Ever	Regular	Regular	Ever	Regular
Never users (*n* = 2221)	0 (0.0%)	49 (2.2%)	1 (0.0%)	15 (0.7%)	185 (8.3%)	13 (0.6%)
Never users who never smoked (*n* = 2172)	–	–	–	14 (0.6%)	149 (6.9%)	13 (0.6%)
Early users (*n* = 649)	46 (7.1%)	182 (28.0%)	6 (0.9%)	38 (5.9%)	268 (41.3%)	26 (4.0%)
Late users (*n* = 419)	–	–	–	17 (4.1%)	117 (27.9%)	10 (2.4%)

At wave 5, rates of regular e‐cigarette use, ever cigarette use and regular cigarette use were higher in late users compared to never users who never smoked (Table 2, right‐hand panel). Logistic regression analyses that controlled for clustering confirmed these differences to be significant when not controlling (regular e‐cigarette use: OR = 6.89, 95% CI = 4.11, 11.54, *P* < 0.001; ever cigarette use: OR = 5.13, 95% CI = 3.85, 6.84, *P* < 0.001; regular cigarette use: OR = 0.34, 95% CI = 1.93, 9.77, *P* < 0.001) and controlling (regular e‐cigarette use: OR = 1.13, 95% CI = 1.04, 1.23, *P* = 0.004; ever cigarette use: OR = 2.87, 95% CI = 2.33, 3.53, *P* < 0.001; regular cigarette use: OR = 1.12, 95% CI = 1.08, 1.16, *P* < 0.001) for covariates.

Table 3 reports the comparison of early versus late users of e‐cigarettes at wave 5 (i.e. 12 or 24 months after reporting first e‐cigarette use for late and early users, respectively). Logistic regression controlling for clustering indicated that there were no significant differences between early and late users for regular e‐cigarette use when not controlling or controlling for covariates (Table 3, left‐hand panel). Similarly, there were no significant differences between early and late users for regular cigarette use when not controlling or controlling for covariates (Table 3, right‐hand panel). In contrast, ever cigarette use was significantly lower in the late compared to early users both when not controlling or controlling for covariates (Table 3, middle panel).

**Table 3:** Prediction of regular e‐cigarette use (left‐hand panel), ever cigarette use (middle panel) or regular cigarette use (right‐hand panel) at wave 5 (15–16 years) by e‐cigarette user group plus covariates (*n* = 1063) controlling for clustering by school

	Regular e‐cigarette use			Ever cigarette use			Regular cigarette useRegular cigarette use		
Predictors	OR	(95% CI)	*P*	OR	(95% CI)	*P*	OR	(95% CI)	*P*
Model 1 without covariates									
Early user	1.00			1.00			1.00		
Late user	0.76	(0.45—1.28)	0.292	0.48	(0.35—0.66)	< 0.001	0.69	(0.35—1.37)	0.282
Model 2 with covariates									
Early user	1.00			1.00			1.00		
Late user	0.96	(0.84–1.11)	0.602	0.61	(0.46–0.81)	< 0.001	0.94	(0.83–1.07)	0.347
Male	1.00			1.00			1.00		
Female	0.89	(0.78–1.01)	0.068	1.72	(1.34–2.21)	< 0.001	0.95	(0.84–1.07)	0.363
Ethnicity = non‐white	1.00			1.00			1.00		
Ethnicity = white	0.84	(0.70–1.01)	0.063	1.08	(0.75–1.55)	0.678	0.91	(0.77–1.07)	0.250
Family affluence	1.00	(0.95–1.04)	0.861	0.98	(0.91–1.05)	0.532	0.98	(0.95–1.02)	0.336
Free school meals = low	1.00			1.00			1.00		
Free school meals = high	0.91	(0.77–1.07)	0.244	1.04	(0.82–1.34)	0.727	1.07	(0.95–1.20)	0.245
Friend smokers = none	1.00			1.00			1.00		
Friend smokers = more than none	1.02	(0.86–1.21)	0.834	0.96	(0.69–1.33)	0.791	0.96	(0.85–1.08)	0.474
Family smokers = none	1.00			1.00			1.00		
Family smokers = one or more	1.02	(0.91–1.15)	0.691	1.11	(0.85–1.46)	0.432	0.91	(0.81–1.04)	0.150
Impulsivity = low	1.00			1.00			1.00		
Impulsivity = high	1.24	(1.11–1.40)	< 0.001	2.22	(1.75–2.82)	< 0.001	1.22	(1.11–1.35)	< 0.001
Intentions = low	1.00			1.00			1.00		
Intentions = high	0.94	(0.78–1.14)	0.538	1.22	(0.82–1.83)	0.314	0.95	(0.75–1.21)	0.691
Attitude = low	1.00			1.00			1.00		
Attitude = high	1.16	(1.01–1.33)	0.034	1.12	(0.81–1.56)	0.468	1.16	(1.03–1.31)	0.020
Perceived norms = low	1.00			1.00			1.00		
Perceived norms = high	1.12	(0.96–1.30)	0.139	0.96	(0.75–1.23)	0.738	1.21	(1.06–1.39)	0.006
Perceived behavioural control = low	1.00			1.00			1.00		
Perceived behavioural control = high	0.86	(0.75–0.99)	0.032	1.05	(0.79–1.39)	0.745	0.97	(0.85–1.12)	0.696
Self‐efficacy = low	1.00			1.00			1.00		
Self‐efficacy = high	1.04	(0.90–1.20)	0.572	1.10	(0.83–1.46)	0.492	0.91	(0.82–1.02)	0.116
Control condition	1.00			1.00			1.00		
Intervention condition	0.93	(0.78–1.11)	0.403	0.73	(0.57–0.95)	0.018	1.01	(0.90–1.13)	0.859

Regular e‐cigarette use: model 1 without covariates, −2 log‐likelihood function = −1474.5; model 2 with covariates, −2 log‐likelihood function = −1208.9; Ever cigarette use: model 1 without covariates, −2 log‐likelihood function = −1507.8; model 2 with covariates, −2 log‐likelihood function = −1440.7; Regular cigarette use: model 1 without covariates, −2 log‐likelihood function = −1515.3; model 2 with covariates, −2 log‐likelihood function = −1180.9. OR = odds ratio; CI = confidence interval.

The comparison of early versus late users of e‐cigarettes when focusing the 12‐month period since first reporting e‐cigarette use (i.e. wave 4 for early users, wave 5 for late users) revealed a slightly different pattern (Table 2). Logistic regression analysis controlling for clustering indicated that regular e‐cigarette use was significantly lower in late compared to early users (OR = 0.54, 95% CI = 0.33, 0.87, *P* = 0.013), although this difference became marginally significant when controlling for covariates (OR = 0.87, 95% CI = 0.76, 1.000, *P* = 0.050). Regular cigarette use was significantly higher in late compared to early users (OR = 1.95, 95% CI = 1.17, 3.25, *P* = 0.012), but this became non‐significant when controlling for covariates (OR = 1.06, 95% CI = 0.96, 1.18, *P* = 0.235). Ever use of cigarettes did not significantly differ between late and early users (not controlling for covariates: OR = 0.89, 95% CI = 0.64, 1.25, *P* = 0.498; controlling for covariates: OR = 1.07, 95% CI = 0.81, 1.41, *P* = 0.625).

## Discussion

The present research shows that, compared to adolescents who had never used e‐cigarettes, those who first reported using them either at age 13–14 (i.e. early users) or 14–15 years (i.e. late users) reported significantly higher rates of regular e‐cigarette use plus ever and regular cigarette smoking at age 15–16 years (Table 2). In early users of e‐cigarettes this pattern was present 12 and 24 months later (this pattern for ever and regular cigarette use in this sample has previously been reported) [5, 7]. In late users of e‐cigarettes this pattern was present 12 months later. These findings add to previous work showing that e‐cigarette use is associated with subsequent ever [1, 2, 3, 4, 5, 6] and regular use of cigarettes [7] and additionally shows that it is associated with subsequent regular e‐cigarette use at age 15–16 years. The current results are comparable to those reported in a recent meta‐analysis of 17 such studies (OR = 4.59, 95% CI = 3.60–5.85) for ever smoking based on comparing never versus ever users of e‐cigarettes [21]. The reviewed studies were generally over a period of 12 months, focused on ever smoking, and controlled for the effects of a number of covariates. Nevertheless, these studies cannot conclusively rule out the idea that other pre‐existing risk factors that were not assessed explain both c‐cigarette and cigarette use (i.e. the common liabilities explanation).

The more novel aspect of the current findings was in assessing differences between early users and late users of e‐cigarettes on subsequent regular e‐cigarette use, ever cigarette use and regular cigarette use at age 15–16 years (Tables 2 and 3). At age 15–16 years, early users were significantly more likely to be ever cigarette users than late users. This replicates previously reported findings [9]. There were no such significant differences for regular e‐cigarette use or regular cigarette use. A problem with these comparisons is that early users had a 24‐month period to start using cigarettes after using e‐cigarettes compared to only 12 months in late users. Our comparison of early and late users 12 months after first reporting using e‐cigarettes (i.e. at age 14–15 years in early users versus 15–16 years in late users) revealed few differences. There were no significant differences in ever use of cigarettes (controlling or not for covariates). Regular e‐cigarette use was significantly higher in early compared to late users, while regular cigarette use was significantly higher in late compared to early users. In both cases these differences became marginally or non‐significant when controlling for covariates.

These findings for early and late users of e‐cigarettes over different time‐periods can be interpreted in different ways. It is notable, however, how similar the two user‐groups are on ever use of cigarettes 12 months after first reported use of e‐cigarettes, and that differences only appear to emerge when the early user group had a longer time‐period to progress to ever use of cigarettes. Regular use of e‐cigarettes and cigarettes use also increased during this period in early users of e‐cigarettes. Future research with adolescents followed‐up for longer periods of time are required to more clearly differentiate the effects of age of first use of e‐cigarettes from time delay to outcome. In addition, future research might usefully explore effects in different age groups (e.g. first reported use of e‐cigarettes before 13–14 or after 14–15 years).

Like many studies, our research provides only limited insights into the mechanism relating ever use of e‐cigarettes to subsequent smoking, meaning that we need to remain cautious in making policy recommendations based on these findings. Since we conducted our work, UK legislation has banned marketing and selling e‐cigarettes to minors and UK agencies are required to enforce age of sale, child‐ and tamper‐proof packaging, display age‐of‐sale signage and health warnings on e‐cigarette packaging [22]. Nevertheless, our findings emphasize the value of regulating the marketing/sale of e‐cigarettes to minors in countries where such measures are not in place.

Our study has a number of strengths, including a large demographically diverse sample, low levels of missing data with replacement based on multiple imputation, assessment of effects over 12–24 months in groups who first reported e‐cigarette use at different ages, exploration of effects on regular e‐cigarette use, ever and regular cigarette use, validated self‐reported smoking measures and exploration of covariates. There are also weaknesses. First, our self‐report measures of e‐cigarette use were not validated against objective measures and all covariates were based on self‐report. Secondly, we did not distinguish types of e‐cigarette use (e.g. delivery method, presence or level of nicotine, presence of other substances) except at the final time‐point. Thirdly, we were unable to differentiate effects due to age of first reported use of e‐cigarettes from time delay to assessment of outcome at a range of ages and time‐points. Relatedly, alternative groupings of e‐cigarette by age may produce different findings. Fourthly, we conducted a series of binary tests, rather than an ordinal model that might allow examination of differences between ever and regular cigarette use (although such comparisons would be limited by the relatively small numbers in these groups).

In summary, this study shows that early versus late use of e‐cigarettes by adolescents is associated with significantly higher rates of ever cigarette use at age 15–16 years. This may, or may not, be attributable to early users having a longer period of time to initiate cigarette use. Further research with a broader age‐range of adolescents over longer periods of time is required.

## Declaration of interests

All authors report receiving grants from the National Prevention Research Initiative during the study. The authors have no conflicts of interest.

## Author contributions

**Mark Conner:** Conceptualization; formal analysis; methodology. **Sarah Grogan:** Conceptualization; methodology. **Ruth Simms‐Ellis:** Methodology. **Lisa Cowap:** Methodology. **Christopher J. Armitage:** Conceptualization. **Robert West:** Formal analysis. **Anna‐Marie Marshall:** Conceptualization. **Kamran Siddiqi:** Conceptualization.
